# The relationship between neighbourhood income and youth mental health service use differs by immigration experience: analysis of population-based data in British Columbia, Canada

**DOI:** 10.1186/s12939-024-02352-8

**Published:** 2024-12-18

**Authors:** Ridhwana Kaoser, Padmini Thakore, Sandra Peterson, Mei-ling Wiedmeyer, Cecilia Sierra-Heredia, Shira Goldenberg, Stefanie Machado, Selamawit Hagos, Elmira Tayyar, Yasmin Bozorgi, M. Ruth Lavergne

**Affiliations:** 1 Faculty of Health Sciences, Simon Fraser University, 515 W Hastings St, Vancouver, BC V6B 5K3 Canada; 2 Centre for Gender & Sexual Health Equity, UBC Faculty of Medicine, 1190 Hornby St, Vancouver, BC V6Z 2K5 Canada; 3 Centre for Health Services and Policy Research, University of British Columbia, 201-2206 East Mall, Vancouver, BC V6T 1Z3 Canada; 4 Department of Family Practice, University of British Columbia, 3rd Floor David Strangway Building 5950 University Boulevard, Vancouver, BC V6T 1Z3 Canada; 5 School of Public Health, San Diego State University, 5500 Campanile Dr, San Diego, CA 92182 USA; 6 Department of Family Medicine, Dalhousie University, 1465 Brenton Street, Suite 402, Halifax, NS B3J 3T4 Canada

**Keywords:** Emigrants and Immigrants, Mental Health Services, Community Mental Health Services, Socioeconomic Factors, Adolescent Health Services, COVID-19

## Abstract

**Background:**

We investigated the relationship between neighbourhood income quintile and mental health service use by immigration experience among youth and explored changes during the COVID-19 pandemic.

**Method:**

We used administrative data to examine mental health service use among youth aged 10 to 24 in British Columbia, Canada, between April 1, 2019, and March 31, 2022. We compared rates of community-based mental health service use, emergency department visits, and hospitalizations and the proportion of involuntary admissions by neighbourhood income quintile and immigration. We used models stratified by immigration to estimate the relationship with income.

**Results:**

Non-immigrant youth used substantially more services than immigrant youth. Service use increased following the pandemic’s start and peaked between January and March 2021. We observed a clear income gradient for community-based service use among both immigrant and non-immigrant youth, but the direction of the gradient was reversed. Service use was highest among non-immigrant youth in lower-income neighbourhoods and lowest for immigrant youth in lower-income neighbourhoods. We observed similar patterns of income gradient for non-immigrant youth for emergency department visits and hospitalization. The proportion of involuntary admissions was higher for immigrant youth.

**Conclusions:**

Mental health service use was substantially lower among immigrant youth than non-immigrant youth, but higher proportions of immigrant youth were hospitalized involuntarily. The reverse income gradient patterns observed for community-mental health service use are noteworthy and suggest significant barriers to accessing preventable care among immigrant youth, particularly those living in lower-income neighbourhoods.

**Supplementary Information:**

The online version contains supplementary material available at 10.1186/s12939-024-02352-8.

## Introduction

The onset of most mental illnesses occurs between childhood and early adulthood [1], and it is well established that earlier treatment can improve prognosis [2, 3] and can mitigate the potential adverse effects of poor mental health (MH) on academic achievements and social and emotional development [4, 5]. Patterns of MH service use are different between immigrant and non-immigrant youth, with studies showing that immigrant youth use fewer MH services than non-immigrants [6, 7]. This has sometimes been interpreted as reflecting a “healthy immigrant effect” with claims of better MH based on treated prevalence from administrative data [6]. Recent findings found inconsistencies in MH trends between survey and administrative data, suggesting that administrative data reflect service access rather than need [8]. Epidemiological studies using validated diagnostic assessment measures found a high prevalence of post-traumatic stress disorder, depression, and anxiety among refugee youth compared to non-immigrant youth [9] and a higher prevalence of depression, anxiety and somatic disorders among immigrants of all ages and immigration classes [10]. When seeking MH care, Canadian immigrants are more likely to consult with psychiatrists (versus other MH professionals), compared to people born in Canada, possibly suggesting a higher severity of mental illnesses and/or gaps in access to family doctors, social workers, counsellors, and psychologists among immigrants [11]. A population-based study in Ontario, Canada, found that recent immigrant youth use of emergency MH services increased over time while MH services in the community (e.g., psychiatrists and family physician offices) decreased [12], further suggesting barriers to receiving preventative and timely care.

The link between economic disadvantages and mental illnesses is well-established for immigrants and non-immigrants [13, 14]. Patterns of MH service use also differ by income. For instance, youth from families of lower socioeconomic statuses are more likely to seek MH care in emergency departments as a first point of contact [15] and for follow-ups [16]. Refugee and immigrant youth in Ontario were more likely than non-immigrant youth to present at the emergency department for MH care without prior use of MH services [15]. Another Ontario study on adults found that living in more deprived neighbourhoods was associated with higher use of primary care and hospital services for non-psychotic mental illnesses, though the association was smaller for recent immigrants compared to long-term residents [7]. Otherwise, studies on the relationship between income, immigration, and the use of MH services are scarce. Changes brought on by the COVID-19 pandemic disproportionately disrupted usual access to MH services among youth living in low-income neighbourhoods [17] and immigrant populations in Canada during this period [18]. In this study, we investigated whether the relationship between neighbourhood income quintile and the use of MH services differs by immigration experience in youth and whether this has changed in the context of the COVID-19 pandemic in British Columbia (BC), Canada.

## Method

### Study design

We used longitudinal population-based linked administrative health and migration data to examine MH service use among youth in BC between 2019/20 and 2021/22 by income and immigration group.

### Study setting

The province of BC is situated on the Western Coast of Canada and includes the city of Vancouver, the third-largest metropolitan city in the country. As of April 1st, 2024, the population of BC was estimated to be around 5,647,467 [19]. While early immigrants to Canada predominantly came from Europe, immigrants to Canada since the 1970s have largely come from Asia and the Caribbean [20]. In the province of BC, approximately 29% of residents were born outside of Canada, with China, India, and the Philippines being the top three countries of birth [21].

While Canada’s public healthcare system provides publicly insured health services to citizens and permanent residents, health coverage varies by province and territory. In BC, people with temporary work permits are not eligible for the provincial health insurance.

### Data

We accessed population-based administrative data, including BC’s Medical Services Plan (MSP) registry file / Central Demographics File, physician payments, hospitalizations, National Ambulatory Care Reporting System (NACRS), Vital Statistics Deaths, Immigration, Refugees, and Citizenship Canada’s (IRCC) Permanent Resident Database [22] and MSP Residency data. These were linked by Population Data BC and provided to the research team with a study-specific unique ID across datasets.

### Study population

We included all youth aged 10–24 years old (as of April 1st in each study year) who were living in BC by March 31st, 2020, and who were alive and registered for MSP in BC for at least 75% of one or more fiscal years between April 1st, 2019, and March 31st, 2022. Using the MSP residency data, we excluded individuals with temporary status who were not present in the IRCC data, including refugee claimants and convention refugees who did not yet have permanent resident status, students, individuals with work permits, visitors, diplomats, and people on working holiday visas, as they may not be insured under the provincial plan, and so their service use would be incompletely captured.

### Study variables

#### Immigration

We identified immigrant youth using IRCC records and included first- and second-generation immigrant youth. Individuals aged 10–24 with an IRCC record were included as first-generation immigrants (i.e., born outside of Canada). We also included individuals aged 10–24 born in Canada but with at least one parent with an IRCC record as second-generation immigrant youth since both first- and second-generation immigrant and refugee youth experience common challenges that can risk their mental well-being, such as discrimination and racism, acculturative stress [23], economic hardships and changes in socioeconomic status [24], food insecurities [25], and inadequate housing [26]. Additionally, first and second-generation immigrant and refugee youth have shared experiences of growing up in an immigrant household with parents experiencing acculturative stress, higher rates of unemployment [27], and occupational segregation [28]. Moreover, acculturative stress experienced by parents can spillover to the family [29, 30] with implications for MH [31], and increase the risk of internalizing (e.g. anxiety, depression) [32, 33] and externalization (e.g., aggression, delinquency) problems in children [33]. Non-immigrant youth included all other individuals who were not in IRCC and whose parents, based on shared MSP contract number within the study years, were not found in IRCC. Prior to creating the comparison groups, we examined service use patterns between non-immigrant youth (youth with no first-degree family record of immigration), first-generation immigrant youth, first-generation refugee and permanent residents’ youth, second-generation immigrant youth, and second-generation refugee and permanent resident youth. We found that service use patterns of first- and second-generation immigrants, refugees, and permanent residents were very similar, while service use by non-immigrant youth was substantially higher than all other groups. For these reasons, we combined first- and second-generation immigrants, refugees, and permanent resident youth into a single group.

#### Neighbourhood income

We determined neighbourhood income quintiles using census dissemination areas of residence based on version 7E of the Postal Code Conversion File (PCCF +). A small number of individuals (< 1%) with unknown neighbourhood income quintile were excluded.

#### Service use

We identified all individuals who had a community-based visit related to MH or substance use using MSP claims to capture visits to family physicians, public health physicians, nurse practitioners, pediatricians, and psychiatrists covered under the provincial health insurance plan. Most other specialized mental health services are not publicly insured and cannot be ascertained in our data. Community-based services would include health care clinics, physician’s offices (including virtual), or home visits, as opposed to hospitals and emergency departments. To identify those who had an emergency department visit related to MH or substance use, we used MSP payment information and the National Ambulatory Care Reporting System (NACRS). NACRS captures 73% of all emergency department visits in BC, and the majority of the remainder is captured in MSP [34]. We captured hospitalization for mental illness, substance, or self-harm using the Discharge Abstract Database, which also flags involuntary admissions under the Mental Health Act (see Supplementary Table 1 for ICD-9 and ICD-10-CA codes used to identify MH and substance use services).

#### Other covariates

Age and administrative sex were obtained from the MSP registration file. The options for “gender” are limited to “M and F” on the MSP registration form. As these are not genders and as it cannot be determined whether legal sex, sex assigned at birth, or gender is reflected, we refer to this variable as administrative sex. We also counted the number of Charlson comorbid conditions in the year of the analysis and the prior year, as having other recorded conditions may shape health services use. We categorized metropolitan areas (census metropolitan areas), small urban areas (census agglomerations) and rural/remote settings (areas with strong to no strong metropolitan influence) using the Statistics Canada Statistical Area Classification Metropolitan Influences Zones.

### Analysis

We reported descriptive statistics on cohort demographic characteristics and plotted the rate of community-based MH visits, MH emergency visits, and psychiatric hospitalizations per person, as well as the proportion of involuntary admissions among individuals with psychiatric hospitalization, in three-month intervals between April 1st, 2019 and March 31st, 2022. We used generalized estimating equations with Poisson distribution and log link to estimate unadjusted and adjusted rate ratios for community-based visits, emergency department visits, and psychiatric hospitalizations and models with binomial distribution and logit link to estimate odds ratios for involuntary hospitalization among people with psychiatric admissions. Models accounted for repeated observations with an autoregressive correlation matrix structure and for annual changes over the course of the COVID-19 pandemic with dummy variables for the study year. Adjusted models included confounders of age, administrative sex, rurality, and number of Charlson conditions as a measure of medical comorbidity. As our objective was to determine if the relationship between income and service use varies by immigration experience, models were stratified by immigration, and we compared rate/odds ratios across neighbourhood income quintiles for non-immigrant and immigrant youth. An additional set of models was not stratified but included immigration experience, income quintile, and an interaction term between the two variables. We controlled for age, administrative sex, rurality, and the Charlson index. Data was analyzed using SAS software, Version 9.4 of the SAS system [35].

## Results

### Demographic characteristics

Table 1 provides the demographic characteristics of 864,407 youth between the ages of 10 to 24 included in the analysis. Non-immigrant youth represented 67.6% (*n* = 584,213) and immigrant youth represented 32.4% (*n* = 280,194) of the total cohort. Compared to non-immigrant youth, larger percentages of immigrant youth lived in neighbourhoods in the lowest income quintile (20.7% vs 16.5%) and in metropolitan areas (89.8% vs 58.3%).

**Table 1:** Characteristics of youth (aged 10–24) in BC by immigration group included in analysis

Characteristic	Non-immigrant	Immigrant	Total
Total number of people, *N* (%)	584213 (67.6)	280194 (32.4)	864407
Age, Mean (SD)	17.5 (4.8)	16.6 (4.6)	17.2 (4.8)
Age, Median (IQR)	18.0 (13.0, 22.0)	16.0 (12.0, 21.0)	17.0 (13.0, 22.0)
Administrative sex, *N* (%)			
F	285353 (48.8)	136080 (48.6)	421433 (48.8)
M	298860 (51.2)	144114 (51.4)	442974 (51.2)
Neighbourhood income quintile (after tax), *N* (%)			
Q1 (lowest)	96230 (16.5)	58122 (20.7)	154352 (17.9)
Q2	99756 (17.1)	63616 (22.7)	163372 (18.9)
Q3	113117 (19.4)	58459 (20.9)	171576 (19.8)
Q4	126741 (21.7)	50561 (18.0)	177302 (20.5)
Q5 (highest)	141890 (24.3)	47448 (16.9)	189338 (21.9)
Missing	6479 (1.1)	1988 (0.7)	8467 (1.0)
1st or 2nd Generation, *N* (%)			
1st		114913 (41.0)	
2nd		165281 (59.0)	
Rurality, *N* (%)			
Metropolitan	340864 (58.3)	251497 (89.8)	592361 (68.5)
Small urban	154797 (26.5)	18537 (6.6)	173334 (20.1)
Rural/remote	82188 (14.1)	8177 (2.9)	90365 (10.5)
Missing	6364 (1.1)	1983 (0.7)	8347 (1.0)
Charlson conditions (two-year lookback), *N* (%)			
0 conditions	528356 (90.4)	257377 (91.9)	785733 (90.9)
1 + conditions	55857 (9.6)	22817 (8.1)	78674 (9.1)
# conditions, Mean (SD)	0.10 (0.33)	0.09 (0.30)	0.10 (0.32)

### Patterns of service use April 2019 to March 2022

**Fig. 1:**
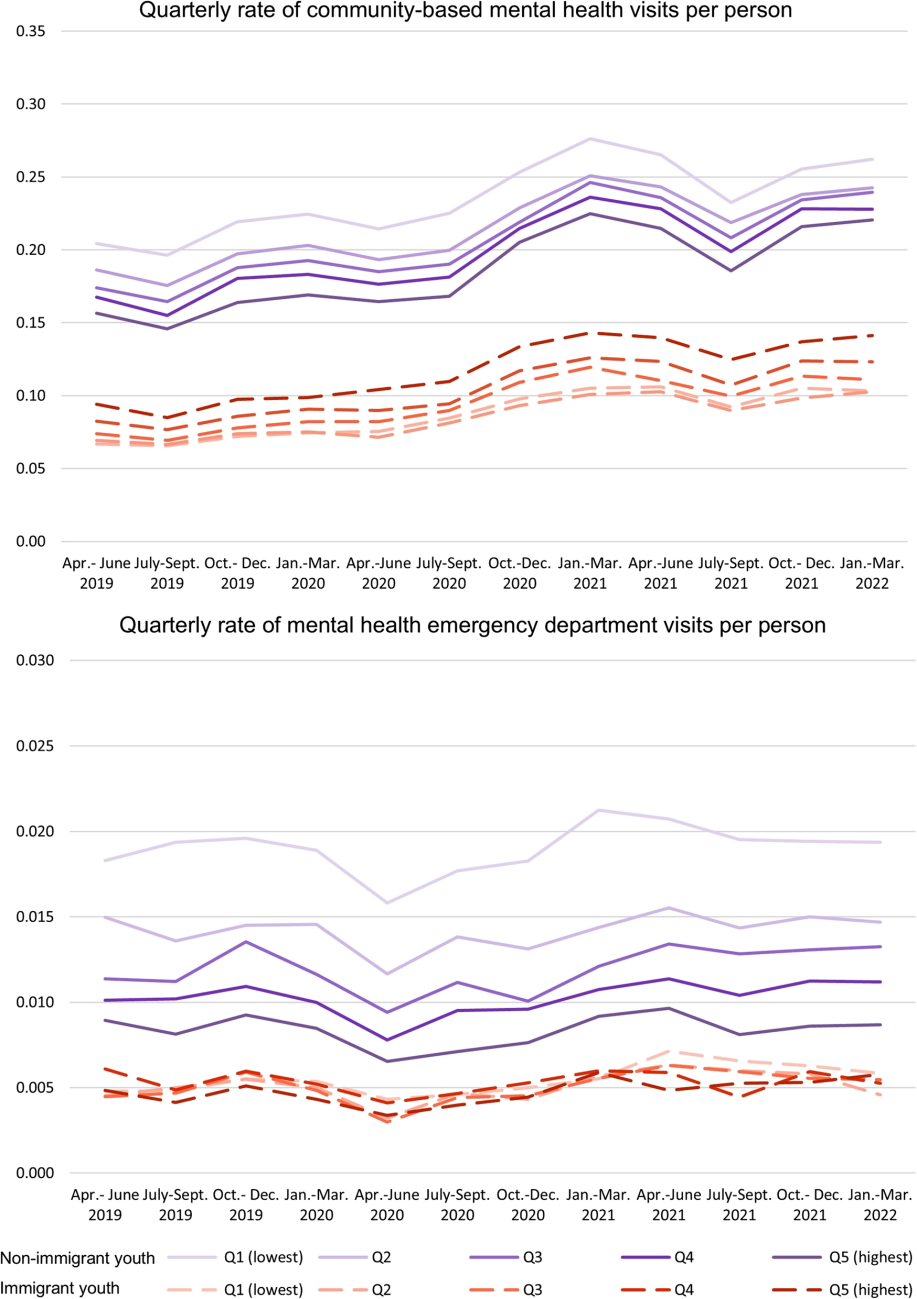
Quarterly rate of community-based mental health visits and emergency department visits per person

The quarterly rates of visits per person for all three types of services were consistently higher for non-immigrant youth than immigrant youth over the study period. Community-based MH service use grew steadily from the start of the pandemic in March 2020 and peaked between January and March 2021 for non-immigrant youth (peak values by neighbourhood income quintile: *Q1 (lowest)*: 0.2762, *Q2*: 0.2510, *Q3*: 0.2461, *Q4*:2361, and *Q5*:0.2247 visits per person) and immigrant youth (*Q1 (lowest)*: 0.1051, *Q2*:0.1008, *Q3*: 0.1197, *Q4*: 0.1260, and *Q5*: 0.1431 visits per person) (Fig. 1). We also observed consistent drops in community-based MH service use for all youth between April and September each year. Emergency service use dropped for all youth shortly after the start of the pandemic between April and June 2020 but increased afterwards. Emergency service use increased slightly over time but was most prominent for non-immigrant youth in the lowest income quintile, peaking between January and March 2021 (*Q1*: 0.0212 visits per person). Among immigrant youth, emergency service use was highest for youth in the lowest income quintile between April and June 2021 (*Q1*: 0.0071 visits per person).

For community-based MH services, we observed a clear income gradient among both immigrant and non-immigrant youth but found that the income gradient was reversed. Rates of visits per person were highest for non-immigrant youth living in the lowest income quintile (Q1) and gradually decreased from Q1 (lowest) to Q5 (highest). In contrast, visit rates among immigrant youth were highest among youth living in the highest income quintile neighbourhoods (Q5) and declined from Q5 (highest) to Q1 (lowest). For emergency department visits, we observed the same pattern of income gradient for non-immigrant youth, with the most visits per person in the lowest income quintile and the fewest visits in the highest. However, Fig. 1 shows no clear patterns of emergency department visits by income quintile for immigrant youth. 

**Fig. 2:**
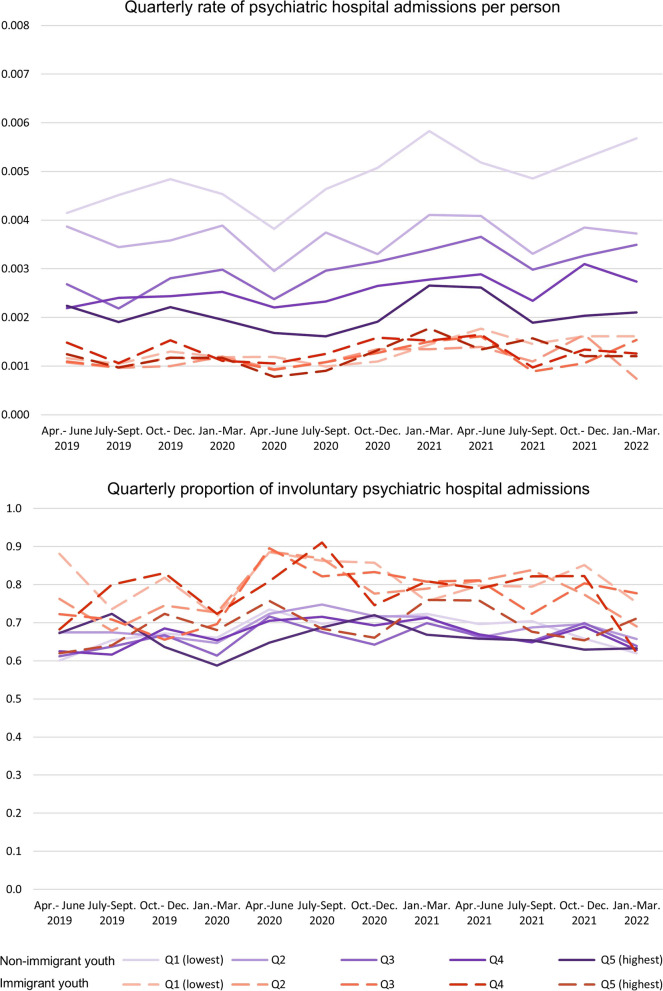
Quarterly rate of psychiatric hospital admissions per person and proportion of involuntary admissions

Figure 2 plots the quarterly rate of psychiatric hospital admissions per person and the proportion of involuntary admissions among those with at least one psychiatric hospital admission by immigration and income quintile. We found that quarterly rates of hospital admission per person increased since the start of the pandemic. Hospital admission increase was most pronounced for non-immigrant youth in Q1 (lowest), Q3, and Q4.

We observed a similar income gradient in the quarterly rates of hospital admissions per person as we observed for community MH and emergency department visits, where non-immigrant youth in Q1(lowest) had the highest rate. No income patterns were observed for immigrant youth with a hospital admission. Overall, immigrant youth from all income quintiles had lower hospital admissions than non-immigrant youth. However, among individuals with psychiatric hospital admission, a higher proportion of immigrant youth were hospitalized involuntarily.

### Relationship between neighbourhood income quintile and service use, by immigration

**Fig. 3:**
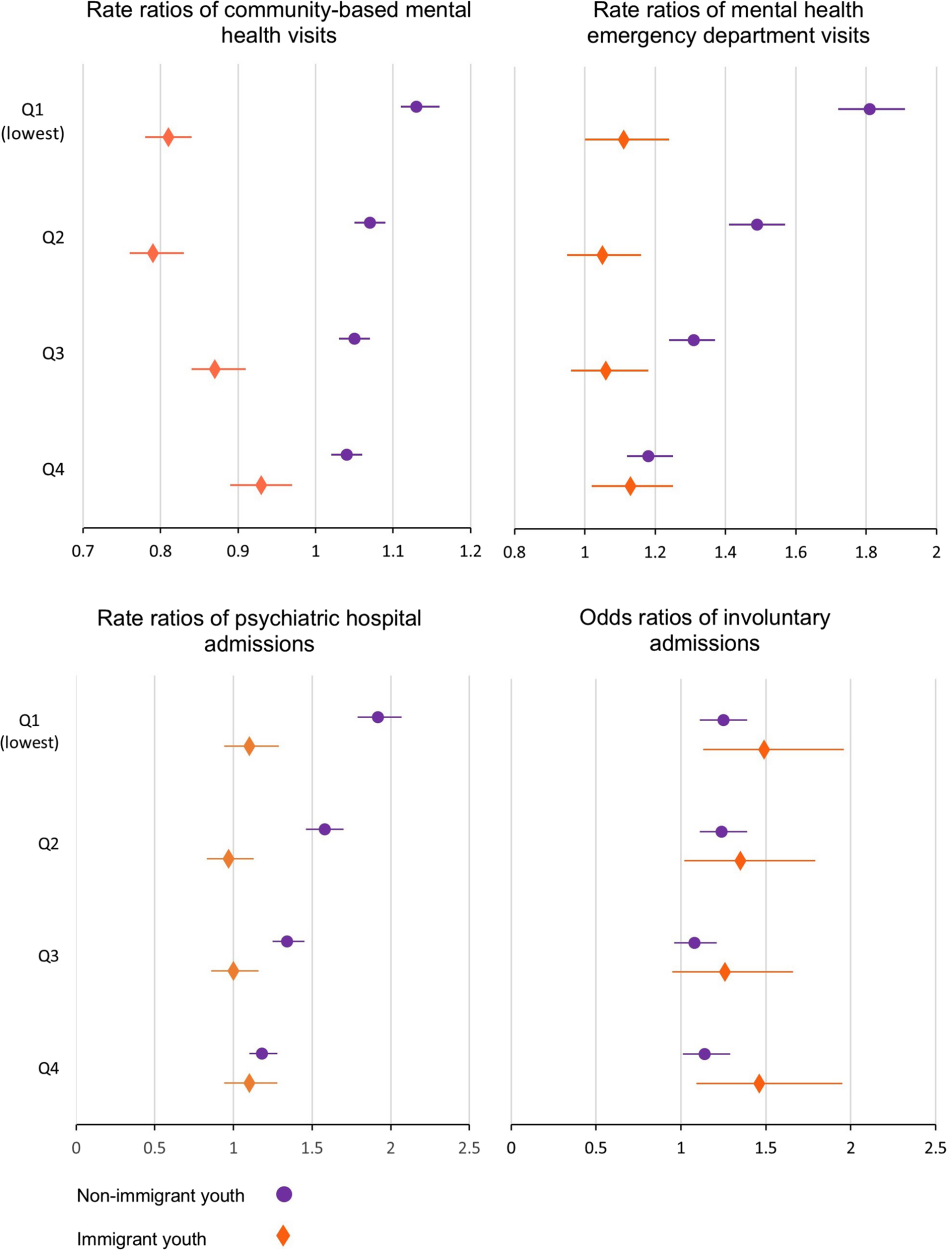
Rate ratios of mental health service use and odds ratios of involuntary admissions

Models adjusting for age, administrative sex, rurality, and multimorbidity (number of Charlson conditions) confirm that the relationship between neighbourhood income quintile and service use differs by immigration. Among immigrant youth, the rate ratios of community MH service use increased with higher neighbourhood income quintile (*Q1 (lowest)*: rate ratio (RR) 0.81, 95% confidence interval (CI): 0.78, 0.84; *Q2*: RR 0.79, 95% CI: 0.76; 0.83, *Q3*: RR 0.87, 95% CI: 0.84, 0.91; *Q4*: RR 0.93, 95% CI: 0.89, 0.97) (Fig. 3, supplementary Table 2). The reverse pattern was observed for non-immigrant youth. Non-immigrant youth in lower neighbourhood income quintiles had significantly higher rate ratios of community-based MH visits than non-immigrant youth in the highest neighbourhood income quintile (*Q1 (lowest)*: RR 1.13, 95% CI: 1.11, 1.16; *Q2:*1.07, 95% CI: 1.05, 1.09; *Q3*: RR 1.05, 95% CI: 1.03, 1.07; *Q4*: RR 1.04, 95% CI: 1.02, 1,06).

Income gradients were similarly observed among non-immigrant youth for emergency department visits (*Q1(lowest)*: RR 1.85, 95% CI: 1.76, 1.94, *Q2:* 1.52, 95% CI:1.44, 1.60, *Q3*: RR 1.33, 95% CI: 1.27, 1.40, *Q4*: 1.19, 95% CI: 1.13,1.25) and hospital admission (*Q1(lowest)*: 1.92, 95% CI: 1.79, 2.07, *Q2*: RR 1.58, 95% CI: 1.46, 1.70), *Q3*: RR 1.34, 95% CI: 1.25, 1.45, *Q4*: RR 1.18, 95% CI: 1.10, 1,28). However, no clear relationship was observed between neighbourhood income quintile and emergency department visits or hospital admissions among immigrant youth.

Among youth who had at least one psychiatric hospital admission, non-immigrant youth in *Q1(lowest)* (OR 1.25, 95% CI: 1.11, 1.39), *Q2* (OR 1.24, 95% CI: 1.11, 1.39), and Q4 (OR 1.14, 95% CI: 1.01, 1,29) had significantly higher odds of involuntary admission than those in Q5 *(highest)*. Immigrant youth from Q1*(lowest)* (OR 1.49, 95% CI: 1.13, 1.96), Q2 (OR 1.35, 95% CI: 1.02, 1.79), and Q4 (OR 1.46, 95% CI: 1.09, 1.95) had significantly higher odds for involuntary admissions than immigrant youth from Q5 *(highest)*. Both immigrant and non-immigrant youth with administrative sex F had higher rate ratios of using all three types of MH services than youth with administrative sex M; however, the odds ratios for involuntary admission were higher for both immigrant and non-immigrant youth with administrative sex M (Supplementary Table 2). Models reveal marked differences by immigration experience and neighbourhood income quintiles for community-based services, emergency department visits, and psychiatric hospital admissions. Models including an interaction between immigration and income confirm these reversed income gradients observed in stratified models (see Supplementary Table S3).

## Discussion

This study found that MH service use in all settings (i.e., community, emergency department, and hospital) was lower among immigrant youth than non-immigrant youth, consistent with existing literature [12, 36]. As reported in prior studies [7, 37], non-immigrant youth in lower-income neighbourhoods interacted more with MH services than those in higher-income neighbourhoods. However, the reverse income gradient observed for immigrant youth for community-based MH visits was unexpected and noteworthy. Given the relationship between poverty and mental illness [38], lower service use among immigrant youth in low-income neighbourhoods observed in our study cannot plausibly be explained by having lower needs, especially when significantly higher service use was found among non-immigrant youth from lower-income neighbourhoods. Other national research has found that immigrants had unmet MH needs [39] and that immigrants are less likely to have an MH consultation than their Canadian-born counterparts, even when reporting poor MH [40]. Our interpretation is that our results point to profound and entrenched barriers to accessing community-based services for immigrant youth, particularly those in lower-income settings.

Barriers to community-based services that might provide earlier intervention and prevent MH crises may also explain our observation that the proportions of involuntary hospitalization were higher among immigrant youth than non-immigrant youth. Our finding is consistent with existing literature that shows that ethnic minorities and refugees experience more involuntary admissions than the general population in Canada [41, 42] and Western Europe [43, 44]. An additional explanation is the involvement of police in MH crisis calls. Evidence from Ontario found that police are 12 times more likely to refer White people to community-based services than people of colour [45] and more likely to coercively admit people of colour to emergency psychiatric services [46].

Observed trends over time showed that the use of community-based MH services increased following the COVID-19 pandemic for all youth, while emergency department visits and hospitalization for MH dropped immediately after the start of the pandemic but increased shortly after. Similar trends were observed in Ontario [47, 48]. This may reflect the closure of facilities and avoidance of care to reduce the risk of COVID-19 infection in healthcare settings. We consistently observed a decline in community-based MH service use for all youth during the summer months across the study years. The uptake of community-based MH service use during the school months may reflect support from teachers and school counsellors in connecting students with community-based services [49, 50].

Interpreting our finding that immigrants in low-income neighbourhoods use fewer MH services requires attention to the fact that recent immigrants are overrepresented in low-income neighbourhoods [51], and newcomers face numerous documented barriers to receiving adequate health care [52]. Evidence shows that recent immigrants and people of lower income in Canada are less likely to be attached to a family doctor [53] and, thereby, possibly less likely to access MH care in the community. A Canadian study revealed that new immigrants have limited knowledge about the role of primary care providers and are unaware they can assist them in their MH care [54].

Our findings with respect to income are in contrast to an Ontario study, which found that adult immigrants in more materially deprived quintiles had more interactions with primary MH care than immigrants in less materially deprived quintiles [7]. One possible explanation for the variation in findings is that the Ontario study grouped long-term immigrants with Canadian-born individuals. In contrast, our study grouped second-generation immigrants with first-generation immigrants due to shared immigrant experiences and cultural views on mental illnesses. The Ontario study also used multiple measures of material deprivation to indicate neighbourhood disadvantage, which included people receiving government transfer payments, unemployment, single-parent families, education level, and individuals living below the poverty line. Our study used neighbourhood income quintiles based on the neighbourhood’s average household income, as individual-level socioeconomic information is not available in our data. These differences in findings highlight how the use of various socioeconomic indicators could result in different outcomes.

Other compounding factors that may lead to underutilization of MH services by immigrant groups, including language barriers, distrust of health systems in destination countries, less knowledge about health services, different recognition of mental illness, and higher stigma in seeking MH services [55]. Prior studies found that a higher degree of cultural identification with the destination country [36] and number of years since immigrating [56] were associated with increased use of MH services, suggesting increased awareness of MH services, MH literacy, and reduced stigma with years spent in the destination country. However, a study of Chinese immigrants in BC revealed that MH services continue to be underused by second-generation Chinese immigrants at moderate to high risk for depression [57], suggesting there are factors other than language proficiency and the number of years in Canada limiting access to MH services. The conceptualization of mental illnesses and approaches to addressing mental illnesses differ among communities and cultures [58] Different cultural conceptualizations of mental illness and treatment practices [59–61] may be passed down to future generations and could explain lower use of mental health services among second-generation immigrants.

This study has several limitations. We cannot capture specific ethnicity or racialization with administrative data. Studies in Western countries found that MH service interactions vary by ethnicity and/or racialization [62–64]. We cannot measure individual-level income or household income with the databases used in this study. Neighbourhood income quintiles may not reflect individual income. Administrative data is limited to capturing publicly covered services. Therefore, we cannot capture psychosocial services paid out-of-pocket, covered by employment insurance, or provided by salaried professionals such as services delivered by psychologists, counsellors, or peer-support workers. The fact that we cannot observe visits to private counsellors or psychologists in our data is a substantial limitation. Access to privately funded services may explain lower use among non-immigrant youth in higher neighbourhood income quintiles. However, it is unlikely that immigrant youth are accessing them at a high enough rate to compensate for lower use of publicly funded services. We could not examine service use among immigrants with temporary and precarious status, including refugee claimants and convention refugees who do not yet have permanent resident status, students, and people with work permits, who may face even more profound barriers to needed care. People in their twenties may arrive with study or work permits, and so this is a limitation of our analysis.

To the best of our knowledge, there are currently no studies comparing the relationship between income and MH service by immigration among youth in the province of BC, Canada. Using population-based linked administrative data allowed us to capture a large cohort and their interaction with community-level services, hospitals, and emergency departments. Future studies should investigate factors that could explain variations in community-based service use by neighbourhood income quintiles among immigrant youth, as they differ from the patterns observed in non-immigrant youth. The immigrant landscape is also changing in Canada, and continued research in this field is needed to update findings with new groups of immigrants entering the country as service use varies by immigrant subgroups.

## Conclusion

We investigated mental health service use by youth in British Columbia in various settings. We found that service use was substantially lower for immigrant youth than non-immigrant youth, and income gradients were reversed for community-based mental health services. The proportions of hospital admissions that were involuntary were higher for immigrant youth than non-immigrant youth, suggesting a lack of preventable mental health services in this population. This evidence suggests that first- and second-generation immigrant youth have significant barriers to accessing community-based services, and these barriers are compounded for youth living in lower-income neighbourhoods.

## Supplementary Information

Supplementary Material 1.

## Data Availability

We used health data holdings from the BC Ministry of Health provided by the Data Stewards that were de-identified, linked and made accessible through Population Data BC. We are not permitted to share the research extract used in this analysis. Access to data provided by the Data Stewards is subject to approval but can be requested for research projects through the Data Stewards or their designated service providers. The following data sets were used in this study: BC’s Medical Services Plan (MSP) registry file / Central Demographics File, National Ambulatory Care Reporting System (NACRS), Hospital Separation File (DAD), Vital Statistics Deaths, Immigration, Refugees, and Citizenship Canada’s (IRCC) Permanent Resident Database and MSP Residency data. You can find further information regarding these data sets by visiting the PopData project webpage at: (https://my.popdata.bc.ca/project_listings/21-046/collection_approval_dates). All inferences, opinions, and conclusions drawn in this publication are those of the author(s), and do not reflect the opinions or policies of the Data Steward.
