# Supplementary material for: The relationship between neighbourhood income and youth mental health service use differs by immigration experience: analysis of population-based data in British Columbia, Canada
Source: Int J Equity Health. 2024 Dec 18;23:270. doi: 10.1186/s12939-024-02352-8 (PMC11658176; doi:10.1186/s12939-024-02352-8)
Supplement: Supplementary file 1 — Supplementary Material 1. [file 12939_2024_2352_MOESM1_ESM.docx]

**Supplementary materials**

**Journal:** International Journal for Equity in Health

**Title:** The relationship between neighbourhood income and youth mental health service use differs by immigration: Analysis of population-based data in British Columbia, Canada

**Authors:** Ridhwana Kaoser^1^, Padmini Thakore^2^, Sandra Peterson^3^, Mei-ling Wiedmeyer^2,4^, Cecilia Sierra-Heredia^1^, Shira Goldenberg^2,5^, Stefanie Machado^2,6^, Selamawit Hagos^2^, Elmira Tayyar^2^, Yasmin Bozorgi^2^, & Ruth Lavergne^6^

1. Faculty of Health Sciences, Simon Fraser University, 515 W Hastings St, Vancouver, British Columbia, Canada, V6B 5K3
2. Centre for Gender & Sexual Health Equity, UBC Faculty of Medicine, 1190 Hornby St, Vancouver, British Columbia, Canada, V6Z 2K5
3. Centre for Health Services and Policy Research, University of British Columbia, 201-2206 East Mall, Vancouver, British Columbia, Canada, V6T 1Z3
4. Department of Family Practice, University of British Columbia, 3rd Floor David Strangway Building 5950 University Boulevard, Vancouver, British Columbia, Canada, V6T 1Z3
5. School of Public Health, San Diego State University, 5500 Campanile Dr, San Diego, California, United States, 92182
6. Department of Family Medicine, Dalhousie University, 1465 Brenton Street, Suite 402, Nova Scotia, Canada, B3J 3T4

**Corresponding Author:**

Dr. M. Ruth Lavergne

e-mail: [ruth.lavergne@dal.ca](mailto:ruth.lavergne@dal.ca)

*Supplementary Table 1. Diagnostic codes for mental and substance use disorders (ICD-9, ICD-10, NACRS)*

| Mental and substance use illnesses included in the study | NACRS  (ICD-10-CA pick-list codes, 1st EDDIAG1 only) | MSP  (ICD-9, 1st only) | Hospitalization  (ICD-10-CA codes, 1st (Most-Responsible) only except for self-harm) |
| --- | --- | --- | --- |
| Schizophrenia spectrum and psychotic disorders | F20.9, F23.9 | 295, 297, 298 | F20–F29 |
| Bipolar disorder | F31.9 | 296 | F30–F31, F34.0 |
| Other mental disorder | F07.2, F48.9, F99, R45.8 | 306, 307 |  |
| Mood/Anxiety | F32.9, F41.9 | 300, 311, 50B | F06.3, F06.4, F32, F33, F34.1, F34.8, F34.9, F38, F39, F40, F41, F42, F45, F53.0, F63.3, F93.0-2, F94.0 |
| Substance use and addiction | F10.0, F10.3, F11.9, F12.9, F14.9, F15.9, F13.9, F16.9, F18.0, F18.9, F19.9 | 291, 292, 303 304, 305, 312.31 | F10–F19, F55, F63.0 |
| PTSD and Acute stress disorders |  | 308, 309 | F43.0, F43.1 |
| Reactive attachment disorder and disinhbited social engagement disorder |  | 313 | F94.1, F94.2 |
| Personality Disorders | F609 | 301, 310 | F60.0-F60.9 |
| Self harm and poisoning |  |  | DIAGX2-DIAGX25 = X60-X84, Y10-Y19, Y28 (includes undetermined) |
| Intellectual disabilities |  | 317, 318, 319 | F70, F71, F72, F73, F79, F88 |
| Communication disorders |  | 315 | F80.2, F80.0, F80.8, F80.9 |
| Autism Spectrum Disorder |  | 299 | F84 |
| Attention-deficit/Hyperactivity disorder (ADHD) |  | 314 | F90.0, F90.1, F90.2, F90.8, F90.9 |
| Conduct disorder | 360 | 312 | F91.1, F91.2, F91.9 |
| Oppositional defiant disorder |  | 313 | F91.3 |
| Intermittent explosive disorder |  | 312 | F63.8 |
| Pica | F50.9 | 307 | F98.3, F50.8 |
| Avoidance/restrictive food intake disorder | F50.9 | 307 | F50.8 |
| Rumination disorder | F50.9 | 307 | F98.2 |
| Anorexia Nervosa | F50.9 | 307 | F50.0 |
| Bulimia Nervosa | F50.9 | 307 | F50.2 |
| Binge-eating disorder | F50.9 | 307 | F50.8 |
| Other feeding or eating disorders | F50.9 | 307 | F50.8, F50.9 |

Notes: NACRS = National Ambulatory Reporting System; MSP = Medical Services Plan

*Supplementary Table 2. Multivariable regression results for mental health service use (rate ratios) and involuntary admission (odds ratios) among youth aged-10-24 in British Columbia, Canada, stratified by immigration experience*

|  | Non-immigrants | | Immigrants | |
| --- | --- | --- | --- | --- |
| Income Level | **Unadjusted** | **Adjusted^a^** | **Unadjusted** | **Adjusted^a^** |
| Community mental health visits (rate ratio) | |  |  |  |
| Intercept Q5 FY 2019, M | | 0.29 (0.28, 0.30) |  | 0.15 (0.14, 0.15) |
| Q1 (lowest) | 1.17 (1.14, 1.19) | 1.13 (1.11, 1.16) | 0.83 (0.79, 0.86) | 0.81 (0.78, 0.84) |
| Q2 | 1.09 (1.07, 1.12) | 1.07 (1.05, 1.09) | 0.81 (0.78, 0.85) | 0.79 (0.76, 0.83) |
| Q3 | 1.07 (1.05, 1.09) | 1.05 (1.03, 1.07) | 0.89 (0.85, 0.92) | 0.87 (0.84, 0.91) |
| Q4 | 1.05 (1.03, 1.07) | 1.04 (1.02, 1.06) | 0.93 (0.89, 0.97) | 0.93 (0.89, 0.97) |
| F (administrative sex) | 1.51 (1.48, 1.53) | 1.51 (1.49, 1.54) | 1.44 (1.40, 1.49) | 1.47 (1.43, 1.51) |
| Fiscal year 2020/21 | 1.21 (1.20, 1.22) | 1.18 (1.17, 1.19) | 1.32 (1.30, 1.34) | 1.28 (1.26, 1.31) |
| Fiscal year 2021/22 | 1.35 (1.34, 1.36) | 1.29 (1.28, 1.31) | 1.51 (1.48, 1.54) | 1.43 (1.40, 1.46) |
| Emergency department visits (rate ratio) | |  |  |  |
| Intercept Q5 FY 2019, M | | 0.00 (0.00, 0.01) |  | 0.00 (0.00, 0.00) |
| Q1 (lowest) | 1.92 (1.83, 2.02) | 1.85 (1.76, 1.94) | 1.13 (1.02, 1.26) | 1.13 (1.02, 1.25) |
| Q2 | 1.56 (1.48, 1.65) | 1.52 (1.44, 1.60) | 1.07 (0.97, 1.18) | 1.06 (0.96, 1.17) |
| Q3 | 1.36 (1.29, 1.43) | 1.33 (1.27, 1.40) | 1.06 (0.96, 1.17) | 1.06 (0.96, 1.17) |
| Q4 | 1.19 (1.13, 1.26) | 1.19 (1.13, 1.25) | 1.11 (1.00, 1.23) | 1.13 (1.02, 1.25) |
| F (sex) | 1.46 (1.41, 1.51) | 1.43 (1.38, 1.48) | 1.32 (1.23, 1.41) | 1.31 (1.22, 1.40) |
| Fiscal year 2020/21 | 0.92 (0.90, 0.95) | 0.91 (0.89, 0.94) | 0.92 (0.86, 0.97) | 0.91 (0.86, 0.97) |
| Fiscal year 2021/22 | 1.08 (1.05, 1.12) | 1.06 (1.03, 1.10) | 1.16 (1.09, 1.23) | 1.13 (1.06, 1.20) |
| Psychiatric hospital admissions (rate ratio) | |  |  |  |
| Intercept Q5 FY 2019, M | | 0.00 (0.00, 0.00) |  | 0.00 (0.00, 0.00) |
| Q1 (lowest) | 2.03 (1.88, 2.19) | 1.92 (1.79, 2.07) | 1.08 (0.93, 1.27) | 1.10 (0.94, 1.29) |
| Q2 | 1.66 (1.53, 1.79) | 1.58 (1.46, 1.70) | 0.96 (0.82, 1.13) | 0.97 (0.83, 1.13) |
| Q3 | 1.38 (1.28, 1.49) | 1.34 (1.25, 1.45) | 0.98 (0.84, 1.14) | 1.00 (0.86, 1.16) |
| Q4 | 1.20 (1.11, 1.30) | 1.18 (1.10, 1.28) | 1.07 (0.91, 1.25) | 1.10 (0.94, 1.28) |
| F (administrative sex) | 1.56 (1.48, 1.64) | 1.51 (1.43, 1.59) | 1.45 (1.31, 1.60) | 1.44 (1.30, 1.60) |
| Fiscal year 2020/21 | 1.04 (0.99, 1.08) | 1.03 (0.98, 1.07) | 1.07 (0.97, 1.18) | 1.06 (0.96, 1.18) |
| Fiscal year 2021/22 | 1.16 (1.11, 1.22) | 1.14 (1.09, 1.19) | 1.19 (1.08, 1.32) | 1.17 (1.06, 1.29) |
| Involuntary admission among individuals with >1 psychiatric hospitalization (odds ratio) | | | |  |
| Intercept Q5 FY 2019, M | | 1.62 (1.09, 2.39) |  | 0.40 (0.14, 1.14) |
| 1 (lowest) | 1.09 (0.98, 1.22) | 1.25 (1.11, 1.39) | 1.69 (1.30, 2.20) | 1.49 (1.13, 1.96) |
| 2 | 1.14 (1.02, 1.28) | 1.24 (1.11, 1.39) | 1.55 (1.18, 2.03) | 1.35 (1.02, 1.79) |
| 3 | 1.03 (0.92, 1.15) | 1.08 (0.96, 1.21) | 1.47 (1.13, 1.93) | 1.26 (0.95, 1.66) |
| 4 | 1.09 (0.97, 1.22) | 1.14 (1.01, 1.29) | 1.52 (1.16, 2.00) | 1.46 (1.09, 1.95) |
| F (administrative sex) | 0.58 (0.53, 0.62) | 0.61 (0.56, 0.66) | 0.47 (0.39, 0.57) | 0.53 (0.43, 0.65) |
| Fiscal year 2020/21 | 1.26 (1.16, 1.36) | 1.28 (1.18, 1.39) | 1.50 (1.24, 1.83) | 1.62 (1.31, 2.00) |
| Fiscal year 2021/22 | 1.05 (0.97, 1.13) | 1.10 (1.02, 1.20) | 1.23 (1.02, 1.50) | 1.33 (1.08, 1.63) |

^a^Also adjusted for age, rurality, and multimorbidities (# of Charlson conditions)

Supplementary Table 3. Multivariable regression results for mental health service use (rate ratios) and involuntary admission (odds ratios) among youth aged-10-24 in British Columbia, Canada, with interactions between immigration experiences and income quintile

| Parameter | Community-based services  (Adjusted interaction, rate ratio) | Emergency department  (Adjusted interaction, rate ratio) | Hospitalization  (Adjusted interaction, rate ratio) | Involuntary hospitalization  (Adjusted interaction,  odds ratio) |
| --- | --- | --- | --- | --- |
| Intercept | 0.28 (0.28, 0.29) | 0.00 (0.00, 0.01) | 0.00 (0.00, 0.00) | 1.35 (0.93, 1.95) |
| Immigrant | 0.57 (0.55, 0.59) | 0.58 (0.53, 0.63) | 0.62 (0.55, 0.71) | 1.03 (0.84, 1.26) |
| Q1 (lowest income) | 1.13 (1.11, 1.16) | 1.88 (1.79, 1.97) | 1.95 (1.81, 2.10) | 1.24 (1.10, 1.38) |
| Q2 | 1.07 (1.05, 1.09) | 1.53 (1.45, 1.61) | 1.59 (1.47, 1.71) | 1.24 (1.10, 1.39) |
| Q3 | 1.05 (1.03, 1.07) | 1.34 (1.28, 1.41) | 1.35 (1.25, 1.45) | 1.07 (0.95, 1.21) |
| Q4 | 1.04 (1.02, 1.06) | 1.19 (1.13, 1.26) | 1.19 (1.10, 1.28) | 1.15 (1.02, 1.29) |
| Immigrant*Q1 | 0.71 (0.68, 0.75) | 0.60 (0.53, 0.67) | 0.56 (0.47, 0.67) | 1.28 (0.96, 1.70) |
| Immigrant*Q2 | 0.74 (0.71, 0.78) | 0.69 (0.62, 0.78) | 0.61 (0.51, 0.73) | 1.18 (0.88, 1.57) |
| Immigrant*Q3 | 0.83 (0.79, 0.87) | 0.79 (0.71, 0.89) | 0.73 (0.62, 0.87) | 1.25 (0.93, 1.67) |
| Immigrant*Q4 | 0.90 (0.86, 0.94) | 0.95 (0.84, 1.06) | 0.92 (0.77, 1.09) | 1.29 (0.96, 1.74) |
| F (administrative sex) | 1.51 (1.48, 1.53) | 1.40 (1.36, 1.45) | 1.50 (1.43, 1.57) | 0.60 (0.55, 0.64) |
| Fiscal year 2020/21 | 1.20 (1.19, 1.21) | 0.91 (0.89, 0.94) | 1.03 (0.99, 1.08) | 1.31 (1.22, 1.42) |
| Fiscal year 2021/22 | 1.32 (1.31, 1.33) | 1.08 (1.05, 1.11) | 1.14 (1.10, 1.19) | 1.13 (1.05, 1.22) |

Also adjusted for age, rurality, and multimorbidities (# of Charlson conditions)
